# Understanding women's sex hormone physiology—A priority in diabetes care

**DOI:** 10.1111/dme.70109

**Published:** 2025-07-17

**Authors:** Céline Isabelle Laesser, Bettina Weber, David Studer, Reem Jalal Alshareef, Lia Bally

**Affiliations:** ^1^ Department of Diabetes, Endocrinology, Nutritional Medicine and Metabolism Inselspital, Bern University Hospital, University of Bern Bern Switzerland; ^2^ Division of Paediatric Endocrinology and Diabetology University Children's Hospital, University of Zurich Zurich Switzerland; ^3^ Children's Research Centre, University Children's Hospital, University of Zurich Zurich Switzerland; ^4^ Graduate School for Cellular and Biomedical Sciences, University of Bern Bern Switzerland; ^5^ University Diabetes Center, King Saud University Medical City Riyadh Saudi Arabia

**Keywords:** diabetes mellitus, type 1, diabetes mellitus, type 2, energy metabolism, gonadal steroid hormones, menstrual cycle, polycystic ovary syndrome

Women experience profound, dynamic changes in sex hormone exposure throughout their lifespan that directly influence diabetes pathophysiology and management. In the pre‐pubertal period, sex hormone levels remain consistently low, but with the onset of puberty, women begin experiencing the remarkable fluctuations characteristic of reproductive biology. The menstrual cycle alone produces 5‐ to 50‐fold variations in oestradiol and progesterone,^1^ while hormonal contraceptives, except for local intrauterine devices, alter these patterns. Pregnancy elevates oestradiol 100‐ to 200‐fold and progesterone 15‐fold,^2^ with assisted reproduction achieving even higher levels. The menopausal transition then precipitates a sharp decline in oestradiol exposure to levels that actually fall below those of age‐matched men. While menopausal hormone therapy can restore sex hormone concentrations approximating premenopausal follicular phase levels, the achieved exposure shows substantial variability depending on formulation specifics, dosage and administration route.^3^ Additionally, body weight changes represent another important modifier of oestrogen exposure, with weight loss consistently associated with decreased oestrogen levels.^4^

Beyond their reproduction functions, female sex hormones, particularly oestradiol, interact extensively with glucose and energy metabolism pathways.^5^, ^6^ Oestradiol enhances insulin sensitivity, increases insulin secretion, improves β‐cell viability, delays gastric emptying and promotes favourable adipose tissue distribution—all collectively promoting glucose homeostasis. These metabolic advantages are further amplified by oestradiol's critical role in modulating energy expenditure and promoting satiety.^5^ While many mechanistic insights originate from preclinical models, human evidence from studies of the impact of menopause and anti‐oestrogenic cancer therapies on diabetes incidence, and investigations on the glycaemic effect of oestradiol replacement (including menopausal hormone therapy) generally corroborate the glycaemic benefits of oestradiol.^7^ In contrast to oestradiol's beneficial effects, progesterone demonstrates a less well‐characterized and predominantly unfavourable metabolic profile, with its characteristic mid‐luteal phase surge consistently linked to decreased insulin sensitivity.^8^ Similarly, both androgenic progestins (frequently utilized in hormonal contraceptives) and states of hyperandrogenism, as clinically seen in polycystic ovary syndrome (PCOS), have been associated with impaired insulin sensitivity.^9^

While sex hormones demonstrably influence diabetes risk and manifestations, this relationship is fundamentally bidirectional, as impaired glucose homeostasis substantially affects the hypothalamic–pituitary–gonadal axis. This is clinically evidenced by the higher prevalence of delayed menarche, menstrual cycle abnormalities, ovulatory dysfunction, subfertility and premature menopause in women with diabetes compared to those without.^10^ A landmark prospective study of menstrual cycles in adolescents demonstrated that those with type 1 diabetes had a sixfold greater risk of menstrual irregularities compared to their non‐diabetic peers, with the risk escalating proportionally with increasing HbA1c levels.^11^ The authors further quantified this relationship, showing menstrual cycle length increased by 5 days for each 1% (11 mmol/mol) rise in HbA1c, powerfully illustrating the intimate connection between metabolic control and reproductive function in type 1 diabetes. While PCOS and type 2 diabetes frequently coexist, clinicians must remain alert to the growing recognition of PCOS in women with type 1 diabetes.

These findings underscore the complex, still incompletely understood interactions between glucose metabolism, insulin signalling and ovarian function involving multiple levels of the reproductive endocrine system. Ovarian insulin receptors, when exposed to supraphysiological insulin exposure, may mediate gonadotropin‐like effects, increasing androgen production.^12^ Hyperglycaemia itself promotes abnormal ovarian folliculogenesis and accelerates follicular apoptosis, likely contributing to the observed cycle abnormalities and earlier menopause in diabetes populations. Glucose and insulin influence reproductive function not only peripherally at the ovarian level but also centrally through hypothalamic–pituitary regulation. Insulin serves as a critical modulator of the hypothalamic–pituitary–gonadal axis, with insulin deficiency known to suppress kisspeptin expression—a key upstream regulator of GnRH secretion.^13^ While less thoroughly investigated, chronic hyperglycaemia may additionally exert direct inhibitory effects on hypothalamic GnRH release. Together, these central and peripheral disruptions likely predispose women with diabetes to the well‐documented spectrum of reproductive abnormalities including hypogonadism, delayed puberty and menstrual cycle disturbances. Clinical epidemiology further reveals the impact of diabetes on fertility potential, with cohort studies demonstrating 24% and 36% reductions in fecundity rates among women with type 1 and type 2 diabetes, respectively, compared to their non‐diabetic counterparts.^14^ Although the increased PCOS prevalence among women with diabetes may contribute to their reproductive challenges, other factors are likely to play a role, underscoring significant knowledge gaps in this area. These clinical uncertainties are further compounded by insufficient research examining the impact of glucose‐insulin homeostasis on assisted reproductive technology outcomes, and reciprocally, the effects of ovarian stimulation protocols on glycaemic control and metabolic parameters in women with diabetes.

In conclusion, women experience remarkable physiological variations in sex hormone exposure throughout their lifespan that exert profound, clinically significant effects on glucose regulation and metabolic homeostasis. The bidirectional relationship between reproductive endocrinology and glucose metabolism creates complex pathophysiological interactions, where dysfunction in either system propagates through both central neural circuits and peripheral target tissues. Despite these fundamental connections, current research paradigms remain disproportionately focused on pregnancy‐related physiology, while other critical hormonal states—including pubertal transitions, menstrual cycle dynamics, contraceptive use, assisted reproduction, menopausal changes and endocrine cancer therapies—have been systematically understudied. Given the substantial clinical consequences of these interactions, advancing our understanding of women's sex hormone physiology across all these contexts must become an urgent priority.

## CONFLICT OF INTEREST STATEMENT

CIL has received a YTCR grant from both the Bangerter‐Rhyner Foundation and the Swiss Academy of Medical Sciences, as well as support from the Siegenthaler Foundation. LB received a grant from the Swiss National Science Foundation (SNSF‐Grant 10000574). None of the authors reports conflicts of interest related to this work.

## Data Availability

Data sharing not applicable to this article as no datasets were generated or analysed during the current study.

## References

[1] Sherman BM , Korenman SG . Hormonal characteristics of the human menstrual cycle throughout reproductive life. J Clin Invest. 1975;55(4):699‐706. 1120778 10.1172/JCI107979 PMC301805

[2] Tulchinsky D , Hobel CJ , Yeager E , Marshall JR . Plasma estrone, estradiol, estriol, progesterone, and 17‐hydroxyprogesterone in human pregnancy. I. Normal pregnancy. Am J Obstet Gynecol. 1972;112(8):1095‐1100. 5025870 10.1016/0002-9378(72)90185-8

[3] Glynne S , Reisel D , Kamal A , et al. The range and variation in serum estradiol concentration in perimenopausal and postmenopausal women treated with transdermal estradiol in a real‐world setting: a cross‐sectional study. Menopause. 2025;32(2):103‐111. 39689249 10.1097/GME.0000000000002459 PMC12147738

[4] Bennett WL , He JH , Michos ED , et al. Weight loss differentially impacts sex hormones in women and men with type 2 diabetes: Look AHEAD Sex Hormone Study. J Clin Endocrinol Metab. 2024;110:e2001‐e2007. 10.1210/clinem/dgae584 PMC12086392 39219157

[5] Mauvais‐Jarvis F , Clegg DJ , Hevener AL . The role of estrogens in control of energy balance and glucose homeostasis. Endocr Rev. 2013;34(3):309‐338. 23460719 10.1210/er.2012-1055 PMC3660717

[6] Mauvais‐Jarvis F , Le May C , Tiano JP , et al. The role of estrogens in pancreatic islet physiopathology. Adv Exp Med Biol. 2017;1043:385‐399. 29224104 10.1007/978-3-319-70178-3_18

[7] Speksnijder EM , Ten Noever de Brauw GV , Malekzadeh A , Bisschop PH , Stenvers DJ , Siegelaar SE . Effect of postmenopausal hormone therapy on glucose regulation in women with type 1 or type 2 diabetes: a systematic review and meta‐analysis. Diabetes Care. 2023;46(10):1866‐1875. 37729504 10.2337/dc23-0451

[8] Valdes CT , Elkind‐Hirsch KE . Intravenous glucose tolerance test‐derived insulin sensitivity changes during the menstrual cycle. J Clin Endocrinol Metab. 1991;72(3):642‐646. 1997519 10.1210/jcem-72-3-642

[9] Wynn V , Adams PW , Godsland I , et al. Comparison of effects of different combined oral‐contraceptive formulations on carbohydrate and lipid metabolism. Lancet. 1979;1(8125):1045‐1049. 86774 10.1016/s0140-6736(79)92949-0

[10] Thong EP , Codner E , Laven JSE , Teede H . Diabetes: a metabolic and reproductive disorder in women. Lancet Diabetes Endocrinol. 2020;8(2):134‐149. 31635966 10.1016/S2213-8587(19)30345-6

[11] Gaete X , Vivanco M , Eyzaguirre FC , et al. Menstrual cycle irregularities and their relationship with HbA1c and insulin dose in adolescents with type 1 diabetes mellitus. Fertil Steril. 2010;94(5):1822‐1826. 19796762 10.1016/j.fertnstert.2009.08.039

[12] Barbieri RL , Makris A , Randall RW , et al. Insulin stimulates androgen accumulation in incubations of ovarian stroma obtained from women with hyperandrogenism. J Clin Endocrinol Metab. 1986;62(5):904‐910. 3514651 10.1210/jcem-62-5-904

[13] Castellano JM , Navarro VM , Roa J , et al. Alterations in hypothalamic KiSS‐1 system in experimental diabetes: early changes and functional consequences. Endocrinology. 2009;150(2):784‐794. 18845637 10.1210/en.2008-0849

[14] Whitworth KW , Baird DD , Stene LC , Skjaerven R , Longnecker MP . Fecundability among women with type 1 and type 2 diabetes in the Norwegian Mother and Child Cohort Study. Diabetologia. 2011;54(3):516‐522. 21170514 10.1007/s00125-010-2003-6 PMC3650679

